# WUSCHEL-Responsive At5g65480 Interacts with CLAVATA Components In Vitro and in Transient Expression

**DOI:** 10.1371/journal.pone.0066345

**Published:** 2013-06-11

**Authors:** Lindsey A. Gish, Jennifer M. Gagne, Linqu Han, Brody J. DeYoung, Steven E. Clark

**Affiliations:** Department of Molecular, Cellular and Developmental Biology, University of Michigan, Ann Arbor, Michigan, United States of America; University of Nottingham, United Kingdom

## Abstract

The CLAVATA (CLV) signaling pathway is essential for shoot meristem homeostasis in Arabidopsis. CLV acts to limit the expression domain of the stem cell-promoting gene *WUSCHEL* (*WUS*). The closely related receptor-kinases CLV1 and BAM1 are key components in this pathway; however, the downstream factors that link the receptors to *WUS* regulation are poorly understood. The Arabidopsis gene *At5g65480* was recently identified as a direct transcriptional target up-regulated by WUS. We have independently identified this gene which we term CCI1 as a CLV1 and BAM1 interacting protein in vitro and in transient expression. CCI1 has phosphatidylinositide-binding activity in vitro and localizes to the plasma membrane in transient expression. Furthermore, CLV signaling components and CCI1 both partition to detergent-resistant membrane microdomains characterized as lipid rafts.

## Introduction

The aerial organs of the adult plant body are reiteratively initiated from a tightly maintained population of stem cells found at the shoot and flower meristems. Each meristem maintains a small number of stem cells in the center, surrounded by the more rapidly dividing and differentiating daughter cells [1]. The shoot meristems maintain a strict balance between proliferation and differentiation of stem cells throughout the life of the plant.

The shoot meristem (SM) in Arabidopsis is composed of three stem cell layers (L1, L2, and L3). Directly beneath L3 stem cells is the Organizing Center (OC) defined by the expression of the transcription factor WUSCHEL (WUS) [2] Current evidence indicates that WUS protein moves from the OC to the overlying stem cell layers to maintain stem cell identity [3], [4].

The components of the CLAVATA signaling transduction pathway act to spatially restrict *WUS* expression. The CLV pathway components include the CLV3 ligand, the leucine-rich repeat (LRR) receptor-kinase CLV1, the LRR receptor protein CLV2, and CRN, a transmembrane kinase-related protein. Mutations in the CLV components result in expanded *WUS* expression and enlarged meristem [5].

In addition, the CLV1-related BAM1, BAM2 and BAM3 proteins fulfill both redundant and unique roles. In the meristem center, the weakly expressed BAM proteins act redundantly with CLV1 to limit meristem size. However, BAM1 and BAM2 are predominantly expressed in the meristem periphery [6]. Loss of BAM receptors results in a reduction in stem cell accumulation [7]. In addition to their complex roles in meristem development, BAM receptors are expressed throughout the plant, and *bam1 bam2* double mutants exhibit pleiotropic developmental defects ranging from seedling lethality, to reduced vascular branching to male sterility [6], [8]. Critically, CLV1 and BAM receptors can cross-complement each other, indicating that the biochemical function of the individual receptors is largely interchangeable.

Several receptor complexes have been identified by various studies using both transient expression and in vivo analysis. The most commonly detected complexes are CLV1 and CLV1/BAM multimers and a complex of CLV2 and CRN [9]–[11]. Higher ordered interactions between CLV1 and CLV2 complexes have only been detected in transient expression.

The ligand, CLV3, is proteolytically processed to release the CLE peptide, which can then bind the extracellular domain of all of the detected receptor complexes [11], [12]. CLV1, BAM1, BAM2 and CLV2 all have nearly identical binding affinities to the mature CLV3 ligand in vitro [11].

There is a conspicuous lack of understanding of signaling intermediates between the known CLV components and WUS. The only known verified signaling intermediates are the phosphatases POL and PLL1. Identified in a suppressor screen of the *clv* mutant phenotype, *pol pll1* double mutants lack all stem cells and aerial tissues phenocopy *wus* mutants [13]–[16]. POL and PLL1 act downstream of CLV1 to maintain *WUS* expression. POL/PLL1 are plasma membrane localized in a fashion dependent on N-terminal myristoylation and palmitoylation [17]. This localization is required for protein function as the mutant phenotype can only be complemented by expression constructs with both of these acylation sites intact. In addition, POL and PLL1 are phospholipid binding proteins whose phosphatase activity is stimulated by PI(4)P.

In this study, we describe a novel protein CCI1 identified through interaction screens with both CLV1 and BAM1. We present evidence of CCI1 receptor interactions when transiently overexpressed in tobacco, plasma membrane localization, phospholipid binding, and membrane microdomain partitioning.

## Results

### Identification of a Novel CLV1-interacting Protein

We performed a protein interaction screen using the yeast Cytotrap system that involves interactions at the yeast plasma membrane (Supporting Figure 1) [18]. Yeast at the restrictive temperature require that hSos (a Ras GEF) localize to the plasma membrane to replace the temperature sensitive cdc25 isoform. hSos was fused to the CLV1 and BAM1 kinase domain and placed into yeast along with cDNA library from Arabidopsis meristem tissue placed behind a N-terminal myristoylation tag to drive plasma membrane localization. Only those yeast with a cDNA-encoded protein that bound to CLV1 or BAM1 would localize the hSos tag to the plasma membrane and survive at the restrictive temperature. The kinase domains used in this screen corresponded to residues 697–980 for CLV1 and 699–1003 for BAM1. Each bait was screened separately. Because CLV1 and BAM1can replace each other’s function in Arabidopsis [6], we hypothesized that proteins interacting with both kinase domains were more likely to represent physiologically relevant partners. We sequenced 32 putative positive lines from yeast with the CLV1 bait protein and 52 lines from yeast with the BAM1 bait (Supporting Tables 1 and 2). Among these positives, two were identified from both CLV1 and BAM1 screens and only one, *At5g65480*, was identified multiple times in both screens. All positives for *At5g65480* were full-length cDNAs, suggesting that interaction with CLV1 and BAM1 required the full-length protein.

**Figure 1:**
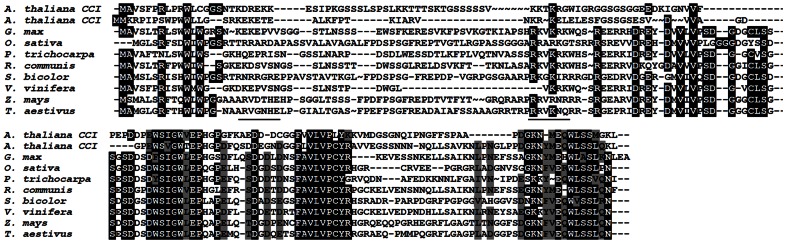
Alignment of CCI1-related proteins from land plants. An alignment of *Arabidopsis thaliana* CCI1, CCI2 and related proteins from various plant species is shown. Conserved residues are shaded at 75%. Basic-rich regions are underlined. The top and bottom segments of CCI1 sequence correspond to the N and C-terminal half constructs used in the lipid-binding assays.

*At5g65480*, which we have named *CCI1* (inspired from *C*lavata *c*omplex *i*nteractor) encodes a small protein of 153 amino acids. While having no known motifs, the genomes of all land plants we analyzed contained homologues of *CCI1* (Figure 1). Arabidopsis contains a second related protein encoded by *At4g38060* which we named *CCI2* (Figure 1). Expression profile mapping of the SM identified *CCI1* as differentially up-regulated in cells of the central zone of the meristem, suggesting an overlap with CLV1 expression and function [19]. Very recently, a screen for direct transcriptional targets of WUS identified *At5g65480* as one of the genes most highly induced by WUS activation. [20].

**Figure 2:**
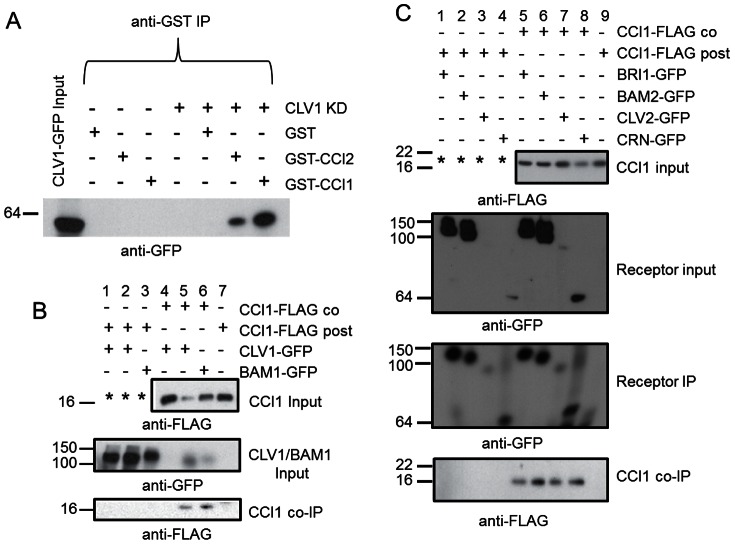
CCI1 interactions with CLV signaling components in vitro and in transient expression. A. Purified CLV1KD-GFP, GST, GST-CCI1 and GST-CCI2 proteins were mixed in various combinations, immunoprecipitated (IPd) with anti-GST antibodies, and the resulting immunoprecipitates were assayed on a protein gel blot probed with anti-GFP. The first lane shows CLV1KD-GFP input. B. Total membrane extracts from *N. benthamiana* leaves expressing CCI1-FLAG and full-length CLV1-GFP and BAM1-GFP IPd with anti-GFP antibodies and co-IP detected with anti-FLAG antibodies. Lanes 1, 2 and lanes 4, 5 are replicates. Note, CLV1-GFP did not express detectably in the lane 4 replicate, nor was there co-IP detected. Experiments represented by lanes 1–3 used an aliquot from the lane 7 expression of CCI1-FLAG alone (*). Co-IP was detected when both CCI1 and BAM1 were co-expressed in the same leaf (CCI1 co), but not when mixed post expression (CCI1 post). C. Total membrane extracts from *N. benthamiana* leaves expressing CCI1-FLAG and full-length BRI1-GFP, BAM2-GFP, CLV2-GFP and CRN-GFP IPd with anti-GFP antibodies and co-IP detected with anti-FLAG antibodies. Experiments represented by lanes 1–4 used an aliquot from the lane 9 expression of CCI1-FLAG alone (*). CoIP was detected when CCI1 and the receptors were co-expressed in the same leaf (CCI1 co), but not when mixed post expression (CCI1 post).

We first tested whether CCI1 directly interacts with the CLV1 kinase domain by expressing the corresponding proteins in *E. coli* as epitope-tagged fusion proteins. In pull-down experiments, GST-CCI1 showed direct interaction with the CLV1 kinase domain, but not in control reactions (Figure 2A). CCI2 also showed direct interaction with CLV1 (Figure 2A).

We next sought to determine whether the CLV1-CCI1 and BAM-CCI1 interactions could be replicated in a plant system. Because efforts to detect epitope-tagged CCI1 expressed in transgenic Arabidopsis were unsuccessful, we used transient expression in *N. benthamiana* to express the proteins [21]. We have successfully used this system to characterize CLV1 interactions both with CLV3 and with other signaling components [11], [22]. To test the interactions between CCI1 and BAM1/CLV1, the full-length proteins were expressed as epitope tagged fusions under the cauliflower mosaic virus 35S cis elements. Two days after infiltration, leaf proteins were extracted and co-immunoprecipitation experiments were performed. When CCI1-FLAG and BAM1-GFP or CCI1-FLAG and CLV1-GFP were co-expressed in the same leaves we detected robust co-immunoprecipitation, suggesting a protein-protein interaction between CCI1-FLAG and the GFP-tagged full-length receptors (Figure 2B).

In addition to GFP-tagged BAM1 and CLV1, GFP-tagged full-length CRN, CLV2 and BRI1 were also tested for interaction with CCI1-FLAG. Unexpectedly, we observed co-immunoprecipitation between CCI1-FLAG and all of the tested proteins (Figure 2C). Co-immunoprecipitations were also detected when the epitope tags were switched (i.e., CCI1-GFP with BAM1-FLAG and CLV1-FLAG) (Supporting Figure 2). Additional control reactions demonstrated that proteins interactions were not a result of non-specific antibody interactions (Figure 2C). Hypothesizing that these associations might be formed spuriously after membrane isolation, we next tested whether the associations of CCI1 with CLV signaling components required co-expression, or could occur by mixing membrane extracts expressing the corresponding proteins. These experiments revealed that co-expression is necessary for any interaction to occur, indicating that the CCI1-receptor interactions were not formed through spurious post-isolation interactions, but instead required that the proteins were expressed simultaneously in the same cells (Figure 2C).

### CCI1 is Plasma-membrane Localized and Binds Phosphatidylinositols in vitro

**Figure 3:**
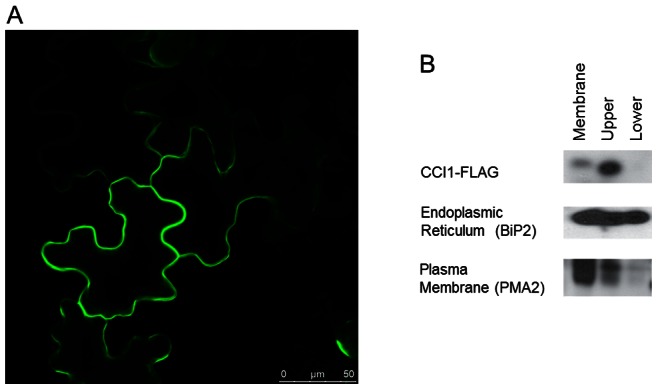
CCI1 is plasma membrane localized. A. Confocal image of CCI1-GFP transiently expressed in *N. benthamiana* leaves 48 hours after infiltration. Signal is detected at cell periphery. B. Two-phase membrane partitioning of CCI1-FLAG transiently expressed in *N. benthamiana* leaves. Endoplasmic reticulum marker BiP2 and plasma membrane marker PMA2 are used to mark the lower and upper phases, respectively.

**Figure 4:**
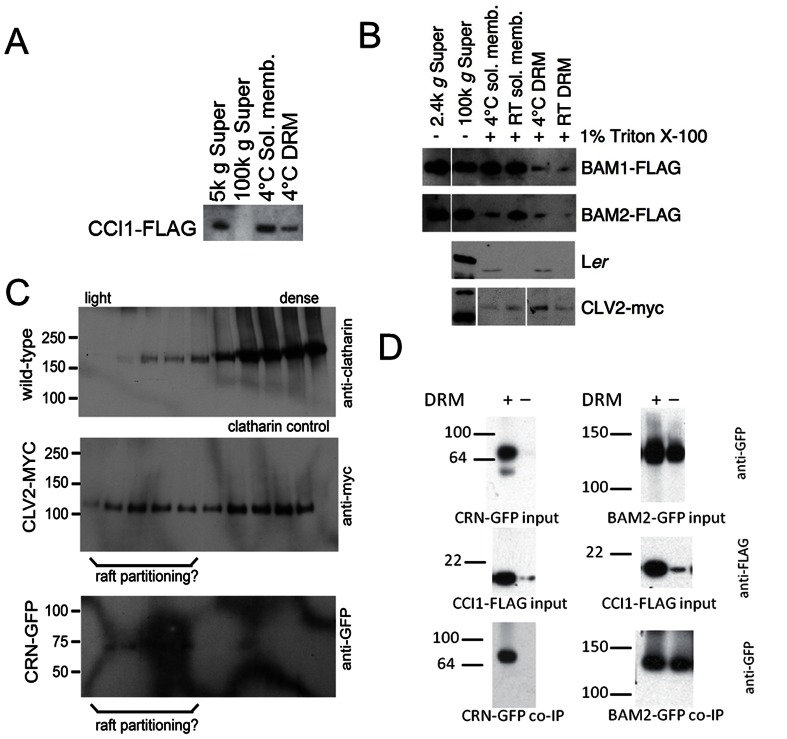
CLV pathway components partition to DRM/raft fractions. A. Partitioning of CCI1-FLAG protein transiently expressed in *N. benthamiana* leaves into soluble membrane and DRM fractions. B. Partitioning of BAM1-FLAG, BAM2-FLAG and CLV2-MYC proteins in stable Arabidopsis transgenic lines into soluble membrane and DRM fractions is shown. C. Sucrose gradient sedimentation of solubilized membrane extracts of clatharin (as a control), CLV2-MYC and CRN-GFP from stable transgenic Arabidopsis meristem tissue. Lipid-associated proteins will float to the lighter fractions. D. Co-IP of transiently expressed BAM2-GFP and CCI1-FLAG was detected in both total membrane fraction (+) and membrane fraction after DRM depletion (–). Co-IP of transiently expressed CRN-GFP and CCI1-FLAG depended on the presence of DRMs.

Because CCI1 interacts with CLV1, which acts at the plasma membrane [23], we next tested whether CCI1 co-localized to the same subcellular compartment. CCI1 has no identifiable localization motif, nor any predicted transmembrane domain. Both CCI1-GFP and CCI1-FLAG were transiently expressed and localization was determined both by confocal microscopy and subcellular fractionation. The localization of CCI1-GFP was consistent with that of plasma membrane localization, with signal exclusively at the cell periphery (Figure 3A). However, the cytoplasm of these cells is largely appressed to the cell periphery, so that we could not exclude partitioning between the membrane and the cytoplasm. To resolve this issue, we fractionated extracts, separating membrane and soluble fractions. For CCI1-FLAG, we detected localization exclusively in the membrane fractions (Figure 4A). In addition, when these *N. benthamiana* leaf protein extracts were subjected to ultracentrifugation and fractionation by two-phase partitioning, CCI1-FLAG was detected in the plasma membrane-enriched PEG phase, and was absent from the plasma membrane-depleted dextran phase [24], (Figure 3B). This localization of CCI1 is consistent with the plasma membrane-localized H+-ATPase PMA2, used as a control. These data collectively indicate CCI1 is plasma membrane bound.

**Figure 5:**
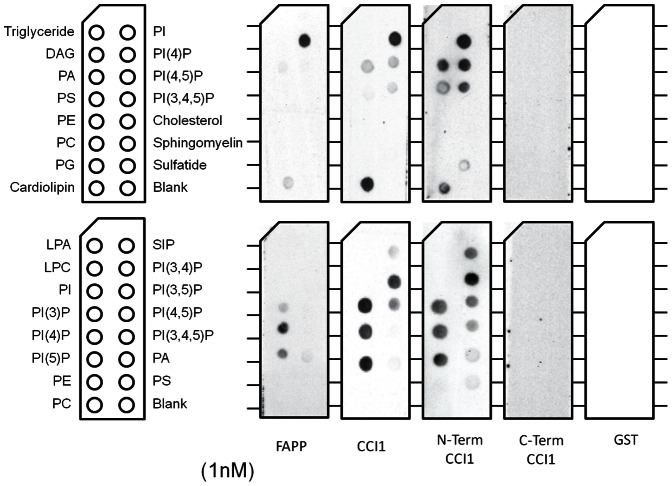
The N-terminal portion of CCI1 binds phospholipids. Echelon membrane lipid strips and PIP strips probed with purified N-terminal GST tagged proteins at a concentration of 1 nM. The PH domain of the human FAPP protein which specifically binds PI(4)P was used as a positive control. The GST tag alone was used as a negative control. N-terminal CCI1 corresponds to the first 83 amino acids of the protein and C-terminal CCI1 corresponds to the remaining 70 (see Figure 1).

These results raised the question of what motif(s) within CCI1 were driving exclusive plasma-membrane localization. As mentioned, neither CCI1 nor any analyzed homologue contains a known membrane-localization motif. Furthermore, CCI1 membrane localization was independent of CLV1 co-expression. One possibility emerged from attempts to use CCI1 as a bait protein in the Cytotrap yeast system. Here we observed that CCI1 alone localized to the yeast plasma membrane (as evidenced by auto-activation, data not shown). As shown previously for the animal protein Tubby, Cytotrap auto-activation can result from lipid binding activity of the bait protein [25]. Furthermore, the CLV1 downstream signaling phosphatases POL and PLL1 autoactivate in the Cytotrap system, localize to the plasma membrane, and bind to phospholipids [17]. To test whether CCI1 has lipid-binding activity, *E.coli* expressed GST-CCI1 was incubated with lipid strips blotted with phosphatidylinositides and other lipids. The human FAPP protein, which has been shown to specifically bind phosphatidylinositol-4 phosphate (PI(4)P), was used as a positive control [26]. Full-length CCI1 bound PI-monophospates and cardolipin, with weak association observed to some PI-di- and tri-phosphates (Figure 5).

Examination of the protein revealed the N-terminal half contains several polybasic stretches of amino acids, conserved across most plant species, while the C-terminal domain has more extensive conservation across land plants (Figure 1). Several phosphatidylinositol-binding domains utilize polybasic patches to interact with negatively charged phosphates on the inositol head group of PI-mono and di-phosphates [27]. When the N- and C-terminal regions were expressed separately as fusion proteins, the N-terminal 83 amino acids of CCI1 were sufficient to bind a similar profile of lipids, while the C-terminal 70 amino acids showed no detectable binding (Figure 5). Deletion constructs targeting individual polybasic regions in the N-terminal portion appeared to attenuate but not abolish lipid binding activity (Supporting Figure 3).

Plasma membranes are not homogeneous with respect to protein and lipid-type distribution [28], [29]. Isolation and visualization of membrane raft microdomains have suggested that specific protein and lipid enrichments in microdomains in the plasma membrane act as hubs to recruit signal transduction pathway components. Some microdomains are sufficiently enriched in sterols, phosphatidylinositols and saturated lipids that they become insoluble to specific detergent treatments [30], [31]. Relative to the total plasma membrane, detergent-resistant membranes (DRMs) are enriched in phosphatydylinositides, such as PI(4)P and PI(4,5)P_2_, over structural phospholipids such as phosphatidylcholine and phosphatidylethanolamine [32]. Furthermore, we have previously observed that CLV3 binding to the CLV1, BAM and CLV2 receptors could only be detected for receptors in DRM fractions, potentially reflecting lipid raft localization [11]. Taken together, we hypothesized CCI1 lipid binding might be associated with membrane microdomain partitioning of as part of signaling complexes.

CCI1 was found in both the soluble membrane and detergent-resistant membrane fractions from *N. benthamiana* transient expression (Figure 4A). CLV2 partitions in a similar pattern in Arabidopsis, while BAM1 and BAM2 are found predominantly in the soluble membrane fraction with detectable partitioning to the DRM fraction (Figure 4B). To test if these receptors were truly localized to lipid rafts, we assayed their sedimentation in sucrose gradients. While the control clatharin was found exclusively in denser soluble membrane fractions, a portion of CLV2 and all detectable CRN from Arabidopsis meristems were found in lighter fractions consistent with lipid raft partitioning (Figure 4C).

The potato sucrose transporter StSUT1 partitions to a DRM fraction of the membrane. Immunoprecipitation of StSUT1 from potato tissue co-immunoprecipitated over 40 associated proteins [33]. This broad array of interactions is thought to result from co-localization to the DRM fraction. In other words, immunoprecipitating a raft-localized protein can pull down the membrane microdomain and all of their associated proteins. Similarly, the co-immunoprecipitation of CCI1 with CLV signaling components could result from their co-localization to DRMs and not necessarily from direct protein-protein interactions. To test this hypothesis, co-IP experiments were performed on both total membrane and DRM-depleted soluble membrane fractions from *N. benthamiana* co-expressing CCI1-FLAG and CRN-GFP, as well as CCI1-FLAG and BAM2-GFP. When the DRM fraction was removed from the membrane fraction, the CCI1/BAM2 interaction was still detectable while the CCI1/CRN interaction was not (Figure 4D). This suggests the interaction between CCI1 and CRN depends on co-localization to the DRM and does not necessarily reflect a direct protein-protein interaction.

### Genetics Analysis of CCI1 Function

We have characterized all three available alleles for *At5g65480*. *cci1-1* is a JIC SM line (GT_5_40258), which contains an enhancer/suppressor-mutator mobile element inserted into At5g65480 [34]. The insertion in *cci1-1* is located 33bp after the start ATG; however, RT-PCR analysis readily detected transcripts from the downstream portion of the gene (Supporting Figure 4). Sequencing the insertion junction revealed that the insertion created an in-frame methionine, leading to a potentially functional protein that was transcribed (Supporting Figure 5). Thus, we conclude that *cci1-1* is not a null allele and may not be hypomorphic. *cci1-1* plants lacked any identifiable mutant phenotype.

*cci1-2* (GABI_541D11) is a GABI-KAT line inserted near the end of the first exon, interrupting the 124^th^ codon, leaving intact the phospholipid binding domain and the conserved domain in the C-terminal portion [35]. Thus, it is not possible to conclude that *cci1-2* is a null allele because the bulk of the coding sequence is left intact. *cci1-2* homozygous mutant plants had no identifiable phenotype.

*cci1-3* (GABI_102G06) is also a GABI-KAT line inserted into the intron between the first and second exons. Homozygous *cci1-3* plants could not be identified in segregating populations from heterozygous parent plants. Sequencing the right border of the T-DNA insertion indicated centromeric satellite sequences, suggesting a possible chromosomal aberration. Analysis of progeny of *cci1-3* heterozygotes indicated a 1∶1 ratio of wild-type to heterozygous plants (32∶30), consistent with lethality due to chromosomal abnormalities. To test this hypothesis, reciprocal crosses were performed between wild-type and *cci1-3* heterozygous plants. Among the F1 progeny, we observed transmission of the *cci1-3* allele through both the male and female gametes. Thus, the failure to observe *cci1-3* homozygous progeny is readily explained by the chromosomal rearrangement associated with the T-DNA insertion, although we cannot rule out the possibility that the *cci1-3* homozygous plants are unviable due to the loss of *CCI1* function.

*cci1-1* was also combined with a T-DNA allele of *CCI2 (*SALK_088916) from ABRC. The double mutant had no identifiable mutant phenotype. In addition, *cci1-1* was also combined with *clv1-11*, *clv2-1* and *clv3-1*. None of the double mutants had an apparent modified meristem phenotype as a result of the *cci1-1* allele (Supporting Figure 6). Whether the lack of interactions reflect the nature of the *cci1-1* allele or the function of the *CCI1* gene is unclear.

## Discussion

We have identified CCI1, a novel CLV1 and BAM1 interacting partner. In this study, CCI1 localized to membranes apparently as a result of phospholipid binding activity. CCI1 partitioned to the plasma membrane, where a significant portion was detected in detergent-resistant microdomains. Consistent with this, CLV signaling components also partitioned to lipid rafts in Arabidopsis. CCI1 not only co-immunoprecipitated with CLV1 and BAM1 when transiently overexpressed in tobacco, but also with other CLV pathway components in a manner dependent on co-localization to the DRM suggesting a role for CCI1 in signaling complexes located in membrane microdomains.

CCI1 is the first protein identified to interact with both the CLV1 and BAM1 kinase domains. CCI1 is a protein with no identifiable domains or motifs. Because prediction programs do not identify any transmembrane domains or lipid modification sites within CCI1, it is likely that the phospholipid binding activity of CCI1 is responsible for its localization. Other studies have shown polybasic regions are sufficient for PIP binding and plasma membrane localization [27]. For example, the C2 domain binds to PIPs by forming a positively charged pocket that interacts with the negatively charged inositiol head group [36]. Interestingly, CCI1 has a very similar in vitro binding profile as the C2 domain of yeast Rsp5p, which is sufficient to drive membrane association [37]. The CCI1 N-terminal PIP-binding domain contains several such basic-rich regions. Although deletion constructs were unable to abolish phospholipid binding activity, it is possible that the multiple, positively charged basic rich regions contribute to electrostatic interactions with the negatively charged phosphate groups.

Because PI(4)P is the only phosphatidylinositol isomer CCI1 binds that is found at significant concentration at the plasma membrane, this is likely to be the PIP CCI1 is binding in vivo. PI(4)P is the most abundant phosphatidylinositol monosphosphate found in plants [38]. PI(4)P is important for PI(4,5)P_2_ synthesis, serving as the substrate for PI(4)P 5-kinase. The POL/PLL1 phosphatases, which act as CLV signaling intermediates, are also plasma-membrane localized and bind PI(4)P, suggesting an important regulatory role for this phospholipid.

The DRM partitioning of CCI1 could be driven by its PI-binding activity and/or protein interactions. DRMs are enriched in sphingolipids, sterols, GPI-anchored proteins and glycerophospholipids, including PIPs, compared to the plasma membrane as a whole [39]. DRMs isolated from *Nicotiana tabacum* plasma membrane are enriched for PIPs when compared to total plasma membrane fractions [32]. Although PI(4)P is found in both plasma membrane and Golgi pools, CCI1 is only detectable in the plasma membrane fraction. In addition, CCI1 is found in both the soluble membrane and the detergent-resistant membrane fractions. This suggests that CCI1 has specificity determinates beyond simply PI(4)P. In addition, CCI1 may traffic between microdomains of the plasma membrane.

Cells use these raft microdomains in pathogen response, protein trafficking and they are the site of signaling hubs in the plasma membrane. Lipid rafts can both concentrate signaling components while at the same time insulating the raft members from negative signaling regulators such as phosphatases [28]. Signal transduction pathways utilizing membrane rafts have been well-characterized in animal immune response and G-protein signaling [28], [40]. Membrane rafts in plants are enriched in proteins associated with signaling including LRR receptor kinases [41]. Auxin signaling and redox systems in membrane rafts in plants have also been characterized [42], [43].

We have presented evidence supporting DRM partitioning and possible lipid raft association for several CLV pathway components, including CCI1. In addition, CLE binding to CLV2, BAM and CLV1 can only be detected in DRM fractions, suggesting CLV signaling depends on receptor localization to membrane microdomains [11]. The expression profiling data compiled by Yadav et al. and the direct interaction of CCI1 with the partially raft-associated kinases CLV1 and BAM1 combined with the co-immunoprecipitation of CCI1 with DRM-associated proteins when transiently overexpressed in tobacco suggest a role for CCI1 in lipid-raft based signal transduction in the shoot meristem.

While we provide evidence of the biochemical properties of CCI1 in transient expression, the physiological role of *CCI1* in the Arabidopsis meristem remains an open question. This is in part due to the lack of a clear *cci1* null allele as well as the lack of phenotypes in the homozygous alleles that can be identified. Independent evidence, however, suggest a role for CCI1 in meristem function. First, *CCI1* is preferentially expressed within the central zone of the meristem, overlapping *CLV1* expression. Second, CCI1 was recently identified as one of the most highly induced gene by WUS activation and identified as a direct WUS target [20]. Interestingly, WUS also represses CLV1 transcription [44] suggesting that there may be several layers to the feedback regulation in the CLV/WUS pathway, including a potential role for CCI1.

## Methods

### Lipid Binding

Sequences encoding CCI1, CCI2 and the last 70 amino acids of CCI1 (C-term CCI1) were amplified from cDNA and inserted into pGEX5X-1 with BamHI and NotI sites. The sequence encoding the first 83 amino acids of CCI1 (N-term CCI1) was inserted using BamHI and SalI sites. The N-terminus GST fusion proteins were expressed in *E. coli* protein expression strain BL21 CodonPlus (Stratagene). The GST-hFAPP expression construct was kindly provided by Erik Nielsen. The expressed proteins were purified using glutathione sepharose (GE Healthcare).

PIP strips and membrane lipid strips were obtained from Echelon Biosciences. The strips were blocked with 3% fatty acid free BSA in PBS-T for 1 hour at room temperature. The expressed proteins were incubated with the blots at a concentration of 1 nM for 1 hour at room temperature. Lipid-protein interactions were detected using a 1∶10,000 dilution of an anti-GST HRP-conjugated antibody (Genscript).

For deletion construct blots, nitrocellulose membrane was blotted with PI(4)P and PE from Echelon biosciences.

### Co-immunoprecipitation and Fractionation of Transiently Expressed and Arabidopsis Proteins

Binary vectors containing 35S:BRI1, CLV1, BAM1, BAM2, CLV2, CRN C-terminal GFP and CLV1 and BAM1 C-terminal fusion constructs, as well as BAM1-FLAG, BAM2-FLAG and CLV2-MYC have been previously described [6], [11], [45]. CCI1-GFP was generated by replacement of the CLV1 coding sequence in the 35S:CLV1-GFP construct. To generate the 35S:FLAG-CCI1 cassette, the CCI1 coding sequence was amplified and cloned into pENTR/D-TOPO to create entry vectors for subcloning into pEarleyGate 202 via LR clonase reaction.

For transient expression, binary vector constructs were transformed into *Agrobacterium tumefaciens* strain GV3101 and infiltrated along with P19, a viral silencing suppressor [21], into *Nicotiana benthamiana* leaves. After 48 hours, proteins were extracted in buffer (50 mM Tris-HCl, 150 mM NaCl, 10 mM EDTA, 10% glycerol, 10 mM NaF, 10 mM NaVO3, 2% plant specific protease inhibitor cocktail (Sigma), 10 ug/ml chymostatin and 2 ug/ml aprotinin). For stable expression lines, 8–10 Arabidopsis meristems were used for protein extraction. Extracts were centrifuged twice at 5,000 *g* for 10 minutes at 4°C to remove flocculate. Supernatants were centrifuged at 100,000 g for 1 hour at 4°C to separate soluble microsomal fractions. The microsomal fractions were then solubilized using 1% triton X-100 with gentle agitation at 4°C.

When immunoprecipitation was performed with anti-GFP antibodies, the antibody was incubated with the solubilized membrane fraction at 4°C for 2 hours, then protein A agarose was added and incubated for an additional two hours. When immunoprecipitation was performed with anti-FLAG antibodies, anti-FLAG M2-Agarose was incubated with the solubilized membrane fractions for 4 hours at 4°C. Agarose was pelleted at 100 *g*, washed three times, and boiled in SDS buffer containing β-ME.

### Sucrose Gradients

Tissue from 10 apices each of BAM1-FLAG, BAM2-FLAG, CLV1-GFP, and CLV2-myc were collected, placed in tubes containing 200 µL of detergent- and glycerol-free extraction buffer (50 mM Tris pH 8.0, 10 mM EDTA, 100 mM NaCl, 5% protease inhibitor cocktail [Sigma, P9599]) on ice, and homogenized as previously described [6]. Homogenized tissue was centrifuged at 2400 *g* for 10 minutes at 4°C. The supernatants were pooled and centrifuged again at 2400 *g* for 10 minutes at 4°C. SDS sample buffer was added to a portion of the supernatant and boiled for 5 minutes. The remaining supernatant was centrifuged at 100,000 *g* for 1 hour at 4°C. SDS sample buffer was added to the 100,000 *g* supernatant and boiled for 5 minutes. The 100,000 *g* pellet was washed with extraction buffer, then resuspended in extraction buffer with 1% Triton X-100 and incubated on ice for 30 minutes and mixed by briefly vortexing every 10 minutes. Sucrose was added to 1.8 M, bringing the volume to 500 µL. Equal volumes of 1.6 M, 1.4 M, and 0.15 M sucrose solutions were layered on top of the 1.8 M sucrose layer containing the isolated detergent resistant membranes. This sucrose step gradient was centrifuged at 100,000 *g* for 15 hours at 4°C. 250 µL fractions were collected from top down (least dense to most dense) and diluted 14 fold with detergent and glycerol-free extraction buffer. Samples were centrifuged at 100,000 *g* for 2 hours at 4°C. The pellet from each fraction was resuspended in extraction buffer with 0.1% Triton X-100 and the samples were boiled for 5 minutes.

### Two-phase Partitioning

The membrane fraction from tobacco leaves transiently expressing FLAG-CCI1 was isolated as described above and resuspended in microsome resuspension buffer containing 330 mM sucrose, 2 mM DTT, 5 mM KH_2_PO_4_, 10 mM EDTA, 10 mM NaF, 10 mM NaVO_3_, 2% plant specific protease inhibitor cocktail (Sigma), 10 ug/ml chymostatin and 2 ug/ml aprotinin, pH 7.8. The plasma membrane fraction was extracted using PEG-dextran phases containing 6.4% (w/w) PEG 3350 and 6.4% dextran (w/w) [24]. Antibodies against plasma membrane marker PMA2 [46] and endoplasmic reticulum marker BiP2 (SPA-818; Stressgen) were used as controls.

### *E. coli* Expressed Protein Co-immunoprecipitation

The coding sequence for the CLV1 kinase domain and C-terminal GFP tag was cloned into pDEST42 and expressed in BL21 codon plus cells. Soluble sonicate from GST-CCI1 or GST-CCI2 and CLV1 KD-GFP were combined, then incubated with glutathione sepharose 30 minutes at room temperature. The sepharose was washed 3 times and eluted. The co-immunoprecipitation was detected with ab6556 (Abcam).

## Supporting Information

Figure S1 **The Cytotrap system uses Ras recruitment to identify protein-protein interactions.** The *cdc25* temperature sensitive mutant is complemented when the human homologue hSos, fused to the bait protein, is recruited to the plasma membrane upon a protein-protein interaction with a myristoylated cDNA library target.
(TIF)

Figure S2 **Solubilized membrane extracts from** ***N. benthamiana*** **leaves expressing CCI1-GFP and full-length BAM1-FLAG and CLV1-FLAG (three replicates in lanes 2–4) were IPd with anti-GFP antibody and the co-IP wasdetected with anti-FLAG antibody.**
(TIF)

Figure S3 **Lipid binding of CCI1 deletion isoforms.** Deletion of several regions of N-terminal CCI1 did not abolish lipid binding activity.
(TIF)

Figure S4 **Reverse transcriptase PCR detected** ***CCI1*** **transcript in the** ***cci1-1*** **allele. Tubulin was used as a control.**
(TIF)

Figure S5 **The** ***cci1-1*** **insertion allele contains an upstream in-frame methionine.** The junction of insertion sequence and *CCI1* coding sequence leads to a possible ORF. *CCI1* coding sequence is in blue. Ds insertion sequence is in red.
(TIF)

Figure S6 **The mean number of carpels per flower in wild-type,** ***cci1-1*** **, and** ***cci1-1*** **combined with mutants of CLV pathway.** Error bars represent standard error of the mean.
(TIF)

Table S1 **Positives from Cytotrap protein-protein interaction screen with the BAM1 kinase domain.**
(DOCX)

Table S2 **Positives from Cytotrap protein-protein interaction screen with the CLV1 kinase domain.**
(DOCX)
