# Supplementary figures and images for: WUSCHEL-Responsive At5g65480 Interacts with CLAVATA Components In Vitro and in Transient Expression
Source: PLoS One. 2013 Jun 11;8(6):e66345. doi: 10.1371/journal.pone.0066345 (PMC3679059; doi:10.1371/journal.pone.0066345)

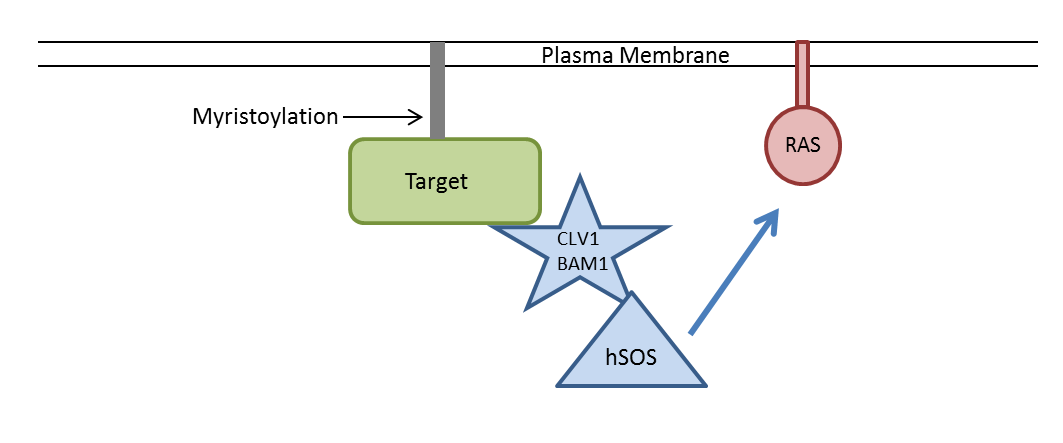

Supplement: Figure S1 — The Cytotrap system uses Ras recruitment to identify protein-protein interactions. The cdc25 temperature sensitive mutant is complemented when the human homologue hSos, fused to the bait protein, is recruited to the plasma membrane upon a protein-protein interaction with a myristoylated cDNA library target. (TIF) [file pone.0066345.s001.tif]

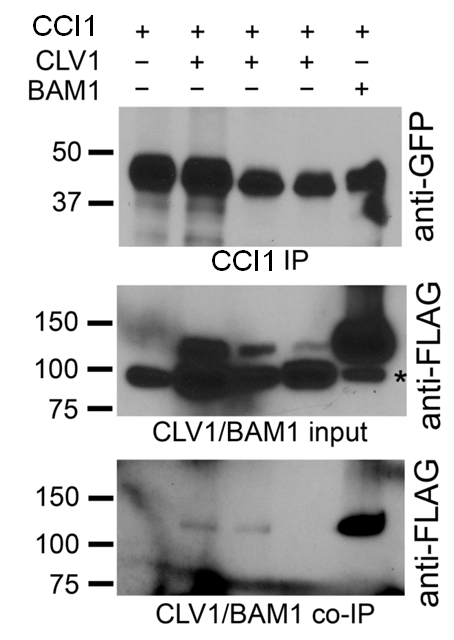

Supplement: Figure S2 — Solubilized membrane extracts from N. benthamiana leaves expressing CCI1-GFP and full-length BAM1-FLAG and CLV1-FLAG (three replicates in lanes 2–4) were IPd with anti-GFP antibody and the co-IP wasdetected with anti-FLAG antibody. (TIF) [file pone.0066345.s002.tif]

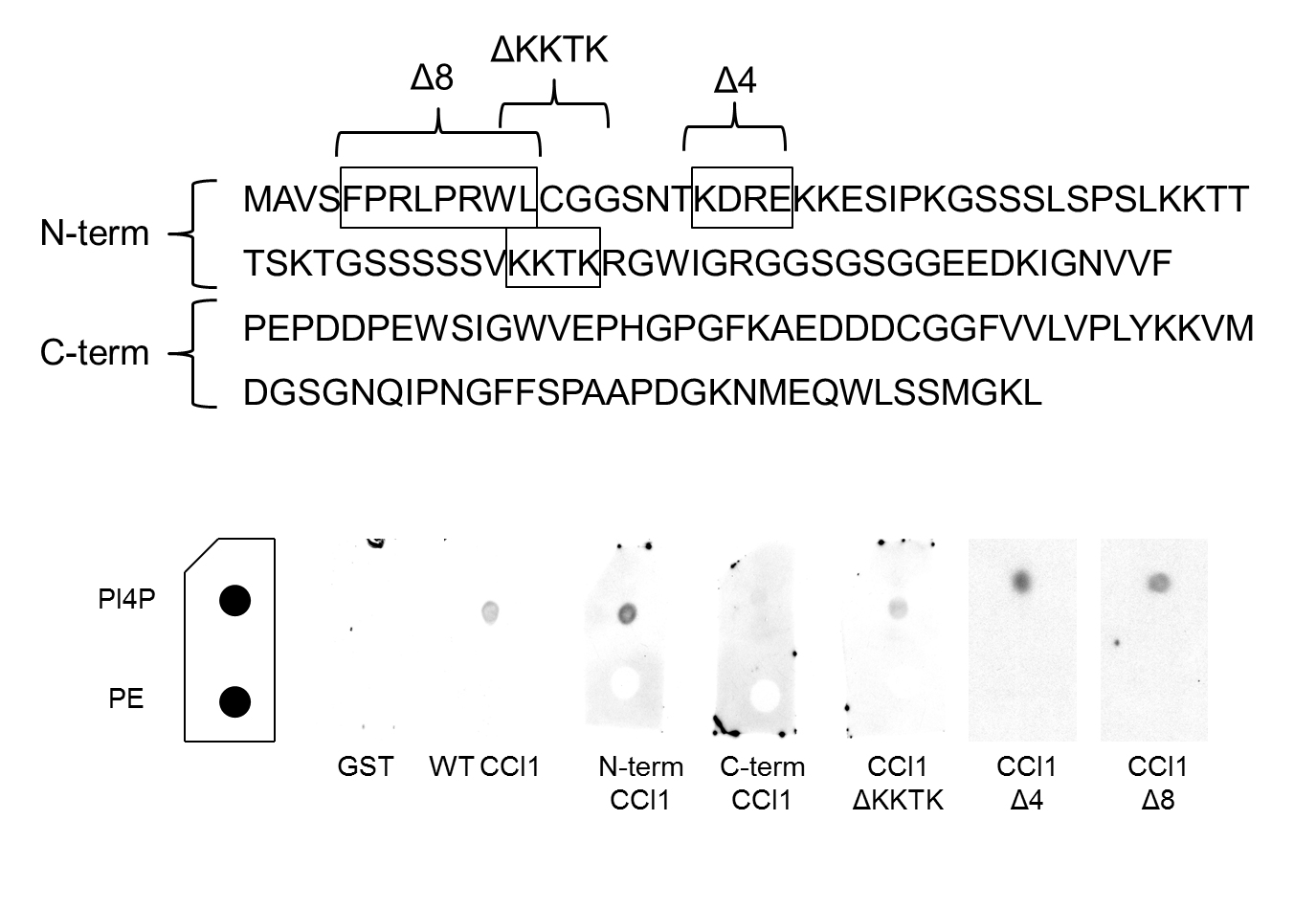

Supplement: Figure S3 — Lipid binding of CCI1 deletion isoforms. Deletion of several regions of N-terminal CCI1 did not abolish lipid binding activity. (TIF) [file pone.0066345.s003.tif]

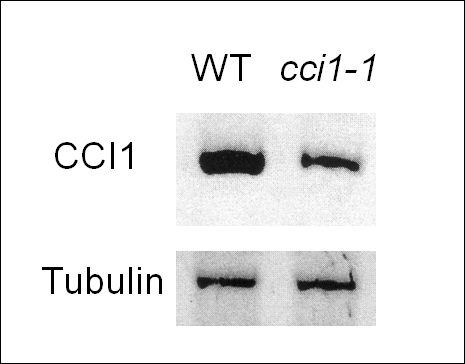

Supplement: Figure S4 — Reverse transcriptase PCR detected CCI1 transcript in the cci1-1 allele. Tubulin was used as a control. (TIF) [file pone.0066345.s004.tif]

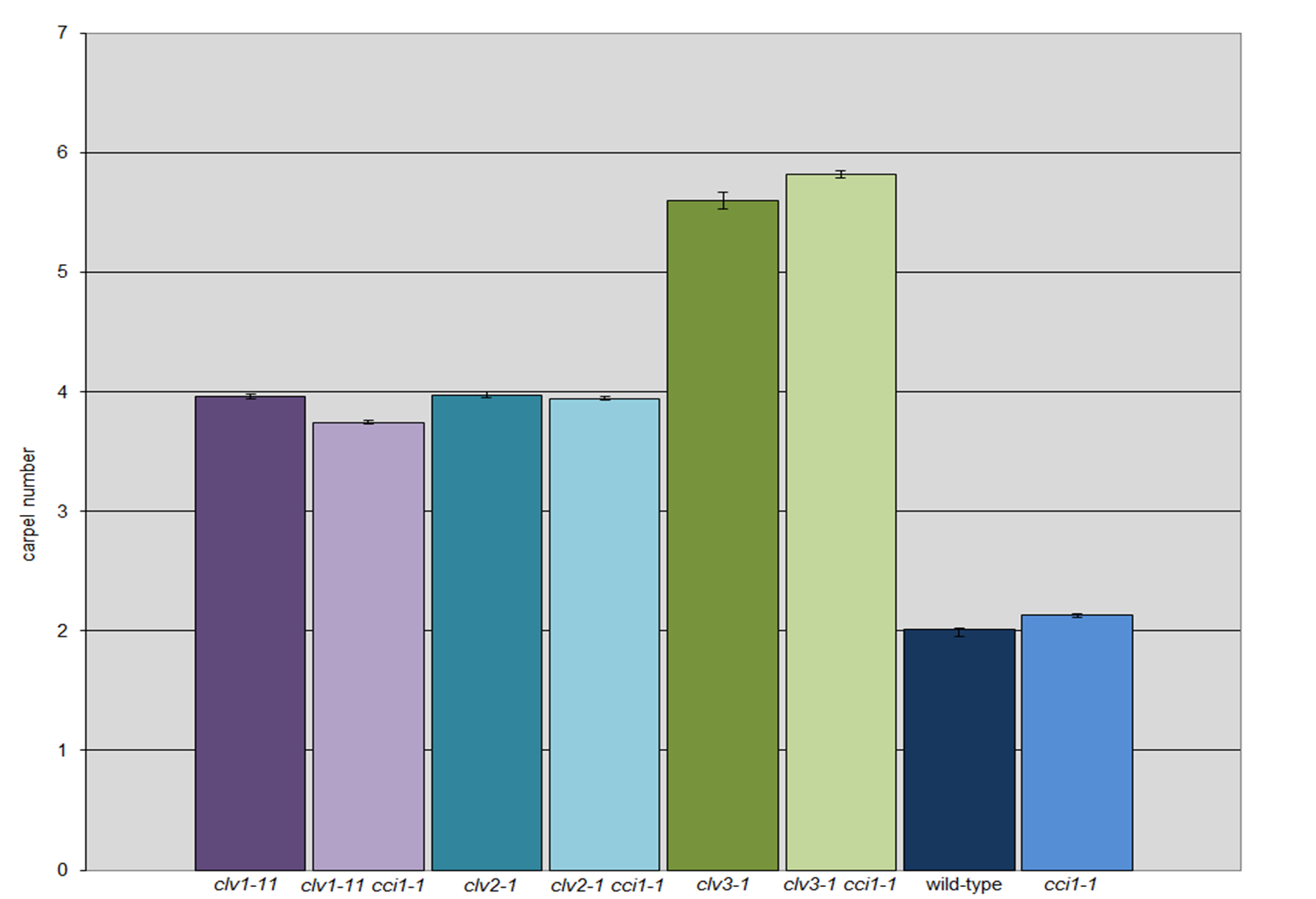

Supplement: Figure S6 — The mean number of carpels per flower in wild-type, cci1-1 , and cci1-1 combined with mutants of CLV pathway. Error bars represent standard error of the mean. (TIF) [file pone.0066345.s006.tif]
