# Supplementary material for: WUSCHEL-Responsive At5g65480 Interacts with CLAVATA Components In Vitro and in Transient Expression
Source: PLoS One. 2013 Jun 11;8(6):e66345. doi: 10.1371/journal.pone.0066345 (PMC3679059; doi:10.1371/journal.pone.0066345)
Supplement: Table S1 — Positives from Cytotrap protein-protein interaction screen with the BAM1 kinase domain. (DOCX) [file pone.0066345.s007.docx]

| **clones** | **Locus Tag** | **Description** |
| --- | --- | --- |
| 5 | AT5G38420 | ribulose bisphosphate carboxylase small chain 2B |
| 5 | AT5G65480 | CCI1 |
| 4 | AT1G20823 | RING-H2 finger protein ATL80 |
| 4 | AT4G34870 | Peptidyl-prolyl cis-trans isomerase CYP18-4 |
| 3 | AT1G49970 | ATP-dependent Clp protease proteolytic subunit-related protein 1 |
| 3 | AT1G52230 | photosystem I reaction center subunit VI-2 |
| 3 | AT2G05100 | photosystem II light harvesting complex protein 2. |
| 3 | AT4G09160 | patellin-5 |
| 3 | AT5G48480 | Lactoylglutathione lyase / glyoxalase I-like protein |
| 3 | AT5G59310 | non-specific lipid-transfer protein 4 |
| 2 | AT1G31330 | photosystem I reaction center subunit III |
| 1 | AT1G07940 | elongation factor 1-alpha |
| 1 | AT1G09140 | ATSRP30 splicing factor |
| 1 | AT1G21830 | hypothetical protein |
| 1 | AT1G55540 | emb1011 Nuclear pore complex protein |
| *1* | *AT2G39730* | *ribulose bisphosphate carboxylase/oxygenase activase* |
| 1 | AT3G02690 | nodulin MtN21 /EamA-like transporter protein |
| 1 | AT3G05900 | neurofilament protein-related protein |
| 1 | AT3G08580 | ADP,ATP carrier protein 1 |
| 1 | AT3G19820 | DWF1 cell elongation protein DIMINUTO |
| 1 | AT3G53430 | 60S ribosomal protein L12-2 |
| 1 | AT4G25050 | ACP4 acyl carrier protein 4 |
| 1 | AT5G17920 | 5-methyltetrahydropteroyltriglutamate--homocysteine methyltransferase |
| 1 | AT5G46550 | DNA-binding bromodomain-containing protein |
| 1 | AT5G54270 | LHCB3 light-harvesting chlorophyll B-binding protein 3 |
