# Supplementary material for: WUSCHEL-Responsive At5g65480 Interacts with CLAVATA Components In Vitro and in Transient Expression
Source: PLoS One. 2013 Jun 11;8(6):e66345. doi: 10.1371/journal.pone.0066345 (PMC3679059; doi:10.1371/journal.pone.0066345)
Supplement: Table S2 — Positives from Cytotrap protein-protein interaction screen with the CLV1 kinase domain. (DOCX) [file pone.0066345.s008.docx]

| **clones** | **Locus tag** | **Description** |
| --- | --- | --- |
| 7 | AT1G08200 | UDP-apiose/xylose synthase |
| 4 | AT5G17770 | cytochrome-b5 reductase |
| 4 | AT5G65480 | CCI1 |
| 3 | AT1G13440 | glyceraldehyde 3-phosphate dehydrogenase |
| 2 | AT2G27020 | proteasome subunit alpha type-3 |
| 2 | AT2G41090 | calmodulin-like protein 10 |
| 2 | AT4G29040 | 26S proteasome regulatory subunit 4-A |
| 1 | AT1G21460 | Nodulin MtN3-like protein |
| 1 | AT1G29930 | chlorophyll a-b binding protein 1 |
| 1 | AT2G07340 | prefoldin 1 |
| 1 | AT2G38450 | hypothetical protein |
| 1 | AT4G28750 | photosystem I reaction center subunit IV A |
| 1 | AT4G38770 | proline-rich protein 4 |
| 1 | AT5G48480 | Lactoylglutathione lyase / glyoxalase I-like protein |
| 1 | AT5G51545 | LPA2 low psii accumulation2 protein |
